# Photography in dermatology

**DOI:** 10.3389/fmed.2026.1854982

**Published:** 2026-08-11

**Authors:** Zarqa Ali, Simon Francis Thomsen

**Affiliations:** 1 Department of Dermato-Venereology and Wound Healing Centre, Copenhagen University Hospital, Bispebjerg, Denmark; 2 Department of Biomedical Sciences, University of Copenhagen, Copenhagen, Denmark

**Keywords:** dermatology, history, images, photographs, teledermatology, telemedicine

## Abstract

Soon after the invention of photography, doctors began experimenting with photographs as a documentation tool. The first known medical photographs were produced in the 1840s and included photographs of skin diseases and facial deformities. Dermatology was one of the first specialties to use photographs, and photography was acknowledged as more objective evidence than the drawings and illustrations used earlier. Photographs have since become an essential part of modern dermatology and are an integrated part of daily clinical practice and patient care. Tips, techniques, and guides to improve clarity, lighting, positioning, exposure, background, angles, and scale are warranted. Three basic elements are essential for high-quality medical photography: resolution, lighting, and focus. Furthermore, three key principles—background, perspective, and angles—are essential for accurately documenting a clinical condition and effectively conveying details about a lesion’s location, size, color, texture, and depth. Using an even, neutral, non-reflecting, monochrome background, positioning the camera perpendicularly at the same level as the skin lesion, and taking multiple shots from different angles are recommended. This study provides a review of good techniques for capturing medical photographs of dermatological diseases for diagnostic use and describes common pitfalls in dermatological photography.

## History of photography

Joseph Nicéphore Niépce, a French inventor, is the creator of the oldest surviving photograph of a view from his window at the Le Gras estate in France. It was created sometime between 1826 and 1827 using a technique called heliography, a process that Niépce had invented around 1822 and that used the hardening of bitumen in light to record an image after washing off the remaining unhardened material (1). In 1835, William Henry Fox Talbot discovered the use of “negatives” in photography with his camera obscura, and he is known as one of the pioneers of modern photography (2).

Soon after the invention of photography, doctors began experimenting with it as a documentation tool. The first mass-produced, commercially available, user-friendly analog camera that made photography a popular activity was launched in 1888 by Kodak, introducing the “Kodak No. 1” camera. The camera used roll film and revolutionized photography (3, 4). In 1975, Kodak built the world’s first digital camera, and decades later, digital cameras became commercially available in the 1990s (5).

At the beginning of the 21st century, there was a technological shift when mobile phones were equipped with a camera (Nokia 7,650, 2002), followed by smartphones and mirrorless cameras. With the iPhone and Android boom in 2007, smartphones took over the market, and more than 90% of all photographs today are taken with smartphones (6).

## Photography in dermatology

The first attempts to create an actual photographic atlas in dermatology were made in 1868 by Alfred Louis Philippe Hardy and M. A. de Montméja. They published “Clinique photographique de l’hôpital Saint-Louis,” containing 50 black-and-white photographs of skin diseases. The majority of these photographs were modified with hand coloring in various shades of intensity to ensure the diagnostic value of the pictures (7). The atlas introduced photography into dermatology and replaced earlier drawn illustrations by providing more realistic descriptions.

In the 1900s, photographs became part of routine dermatological documentation with the development of cameras. The photographs were used to record disease progression, the effect of treatment, and to teach medical students. Dermatology departments began to create archives of thousands of clinical photographs (8, 9). Oregon’s telemedicine application program in 1995 was an early conceptual starting point of teledermatology (10). Photographs have since become an essential part of modern dermatology and are important for documentation, publication, and teaching.

## Methods

A literature search was performed in PubMed from January to March 2025 to identify relevant publications on medical photography in dermatology, and the search was repeated in July 2026. The search was carried out using the following terms: (dermatology or skin disease) and (photography or imaging or teledermatology or smartphone photography). The search included articles published in English; a total of 115,652 records were identified. Additional relevant publications were identified through manual screening of the reference lists of selected articles.

## Technical aspects of dermatological photographs

Photographs have become an important part of everyday clinical practice in dermatology. They are used in referral pathways, documentation, communication among doctors, follow-up regimens, and remote consultations with patients using email or video consultations (11). The improving quality of smartphone photographs has changed dermatologic photography practices, and smartphone photographs are perceived to be comparable to DSLR photographs for use in skin assessments (12). Therefore, a fundamental understanding of medical photography and its principles has become essential for many clinicians, not only to capture high-quality clinical photographs themselves but also to guide their patients in doing so. This may improve the quality and utility of the photographs in a clinical setting.

Many contemporary smartphones support RAW image capture, a digital data file regarded as a “digital negative” that preserves minimally processed sensor data and allows greater flexibility for postprocessing (e.g., white balance, exposure, and contrast adjustment) while minimizing loss of image information. However, RAW files generate larger file sizes and require specialized processing software, making them more suitable for research settings than for routine use in dermatologic practice (13).

### Photograph quality

To assess the dermatological condition via a photograph, the quality of three basic elements—resolution, lighting, and focus—is essential (Table 1).

**Table 1:** Overview of technical parameters and photography techniques to consider when capturing a photograph of a skin lesion for medical purposes.

Category	Recommendation
Camera resolution	Use a camera with a minimum resolution of 5 MP (modern smartphones typically exceed this).
Image format	JPEG is appropriate for routine clinical practice; RAW images may be considered for research applications.
Lighting	Capture images in a well-lit environment using indirect natural daylight whenever possible.
Light source	Avoid direct sunlight and mixed lighting (daylight plus artificial light). Maintain consistent lighting conditions.
Flash	Avoid flash when possible; use it only if ambient lighting is insufficient.
White balance	Use automatic white balance under standardized lighting; include a plain white background when possible.
Focus	Ensure the lesion is sharply focused using autofocus or manual focus if necessary.
Background	Use a plain, non-reflective white, gray, or blue background.
Patient preparation	Remove clothing, jewelry, and hair covering the lesion.
Perspective	Position the camera perpendicular to the lesion with the lesion centered in the frame. Include sufficient surrounding anatomy for orientation.
Photographing distance	Maintain a distance of approximately 10–20 cm for close-up images.
Clinical image set	Obtain both an overview image and a close-up image; include a measurement scale when lesion size is clinically relevant.
Photography angles	Multiple shots from different angles may be required. Photographing the patient in anatomical position is preferred unless the skin lesion of interest is hidden in that position.

#### Resolution

Resolution is the level of detail contained in a photograph, given as the number of pixels, short for “picture element,” the smallest unit, a tiny square or dot, that represents a single point of color that makes up the photograph. A high-resolution photograph contains a large number of pixels, resulting in a more detailed and sharper image (14). A minimum of 5 megapixels (MP) is believed to be sufficient for clinical photography (14–16). The average resolution of smartphone cameras has increased from 12–20 MP in 2020 to more than 50 MP today and is considered adequate (17).

#### Lighting

Lighting is an important factor for obtaining a high-quality photograph, as small changes in lighting can change the appearance of skin features, compromise the correct colors and details, and make diagnosis difficult. Ensuring adequate lighting may improve the quality of the photograph significantly. Light sources with consistent color temperature and a high color rendering index, a quantitative index measuring a light source’s ability to reveal the true colors of objects compared with natural sunlight, provide more accurate reproduction of skin tones and lesion colors (18). It is recommended to capture photographs in a well-lit environment or near a window with ample indirect natural light, as natural sunlight is preferred when capturing medical photographs (19–22). Direct sunlight and mixed lighting sources, such as a combination of daylight with artificial room lighting, should be avoided; maintaining consistent location and orientation relative to the window is preferable. The use of flash light to capture medical photographs is generally not recommended, as it may wash out subtle skin conditions, whitewash darker skin tones, cause sharp reflections and uneven lighting, reduce contrast, decrease fine details, and cast harsh highlights and shadows (14, 23). However, in some situations with limited light or dimness, the use of flash may improve the visibility of certain skin lesions. Ceiling LED panels are preferred over ring-type electric flashes as they have a larger surface area and can cast a graduated soft shadow, minimize glare, and provide uniform lighting (14, 24).

In addition to lighting, white balance (WB) should also be considered, as color fidelity directly influences the assessment of, e.g., erythema, pigmentation, and vascular lesions. WB is the process of adjusting a camera so that white objects appear white under different lighting conditions and thereby removes unrealistic color casts. While the majority of cameras default to an automatic WB setting, the algorithms of which vary by device, using a plain white background under natural daylight can help optimize the camera’s automatic WB. This ensures a more accurate color temperature and minimizes unwanted color casts. Auto WB performance may vary in mixed or inconsistent lighting; however, contemporary smartphones have improved substantially and are generally adequate for routine clinical photography under standardized lighting conditions (25, 26).

#### Focus

Accurate focus is crucial for clinical documentation as blurred photographs can obscure details, making documentation or diagnosis unreliable. Sharp and non-blurry photographs can be achieved with correct focusing. When a skin lesion is in focus, its details will be well-defined, in contrast to out-of-focus photographs, which create soft or blurry images with reduced depth perception and draw attention elsewhere. The majority of cameras have autofocus, and generally, autofocus is adequate for medical photographs (27).

### Photography techniques

A good medical photograph is based on its ability to accurately document a clinical condition and convey information such as lesion location, size, color, texture, and depth/infiltration. Patient preparation is important prior to capturing a medical photograph. Articles of clothing and jewelry should be removed, and hair should be moved away from the area of interest to minimize distractions in the photograph.

#### Background

**Figure 1:**
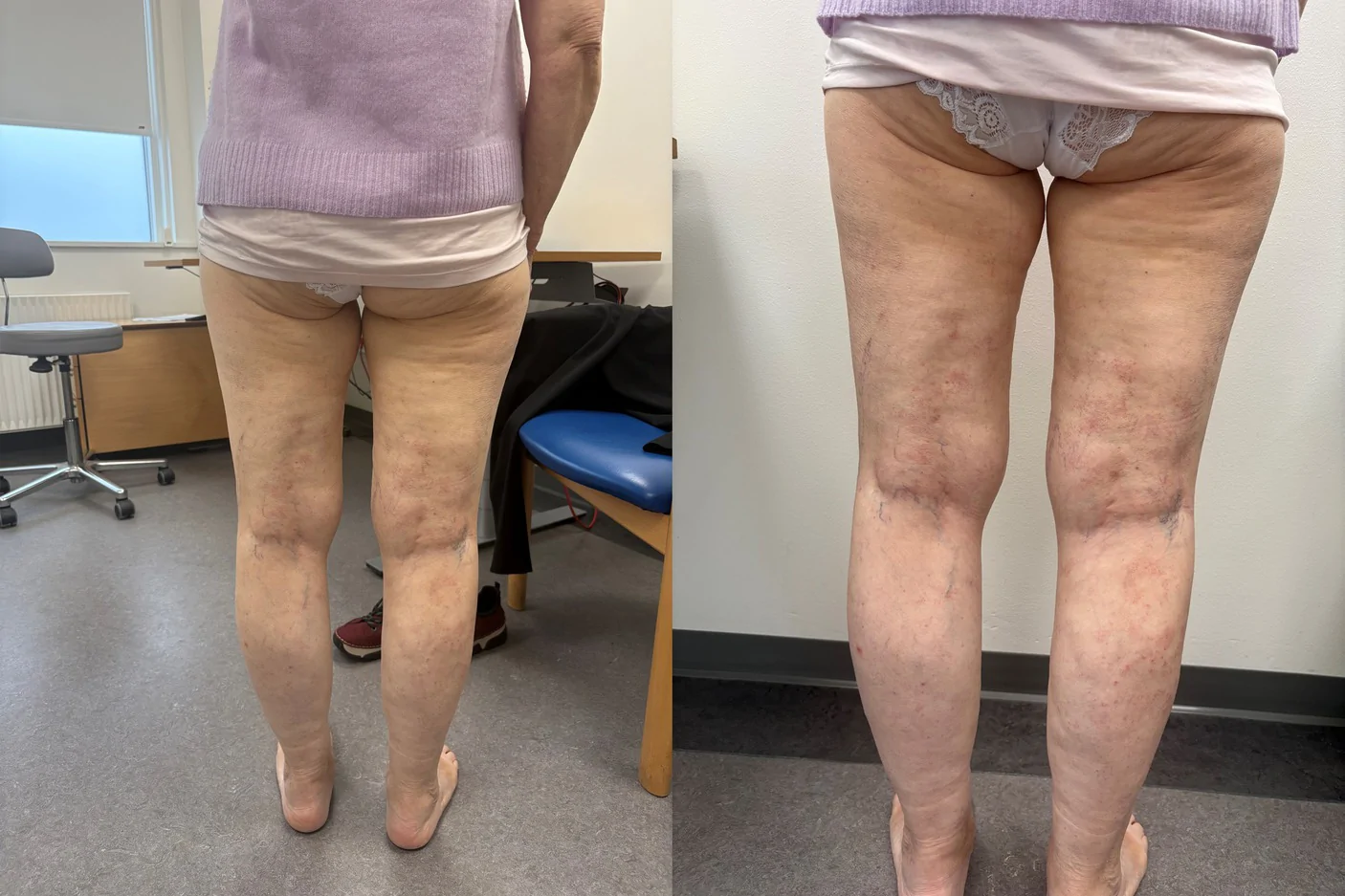
Use a plain, clean background and remove distracting elements from the background (right). Position the patient against a white wall or a monochrome door in the clinic (left).

Attention to background choice is important to minimize distraction and ensure that focus remains on the skin lesion. An even, neutral, non-reflecting, monochrome background is preferred. White, gray, and blue backgrounds are ideal as they provide the most natural skin tones (16, 28). Brightly colored and patterned backgrounds should be avoided as they may alter the appearance of the skin lesion, giving a “warmer” or “cooler” tone, leading to inaccurate color reproduction. In the clinic, a white wall or a monochrome door is ideal as a background (Figure 1).

#### Perspective

**Figure 2:**
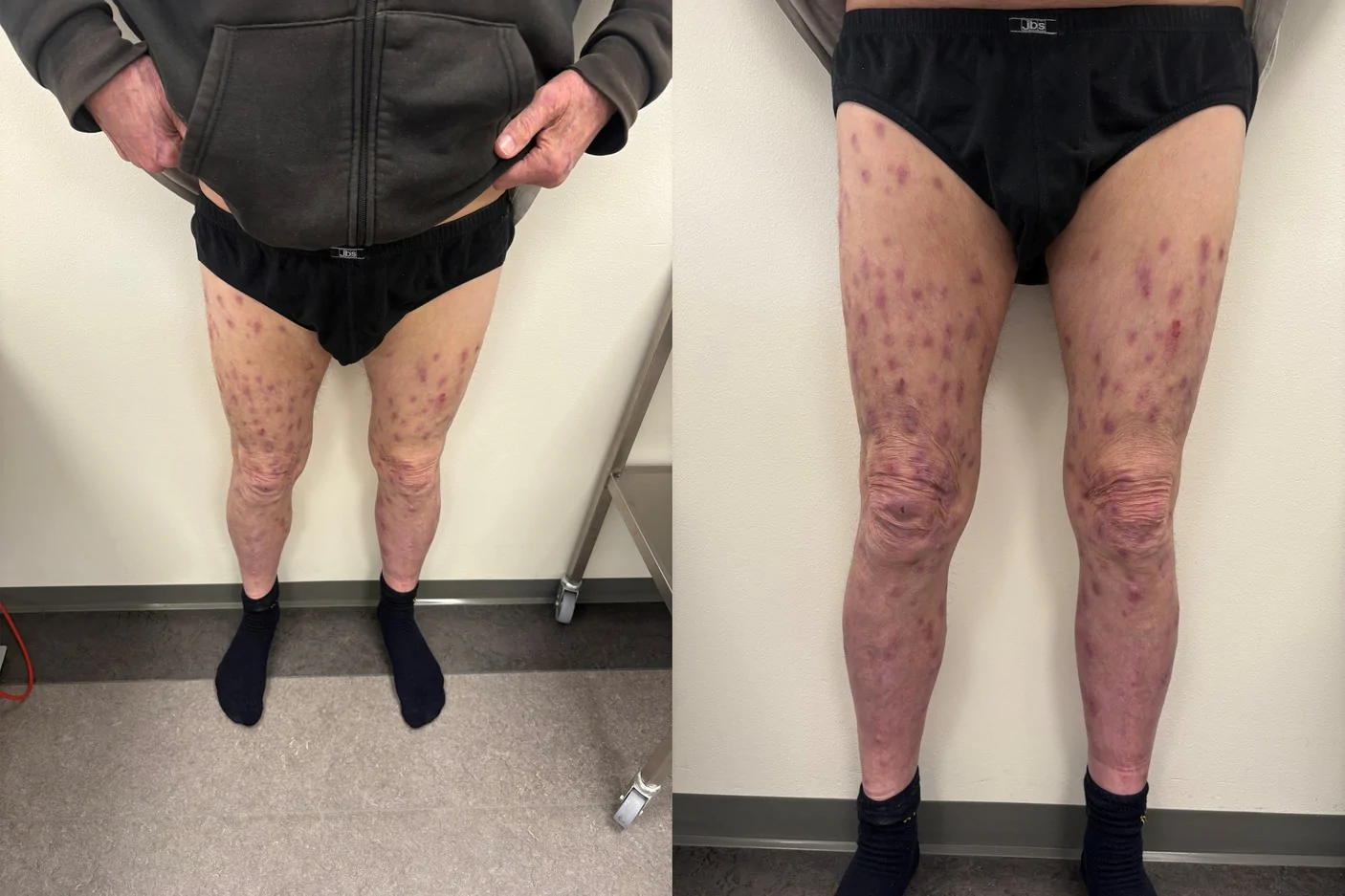
Representative examples of appropriate and inappropriate camera positioning. The camera should be held perpendicular to the skin lesion or anatomical region (left) and should not be angled downward when capturing clinical photographs (right).

**Figure 3:**
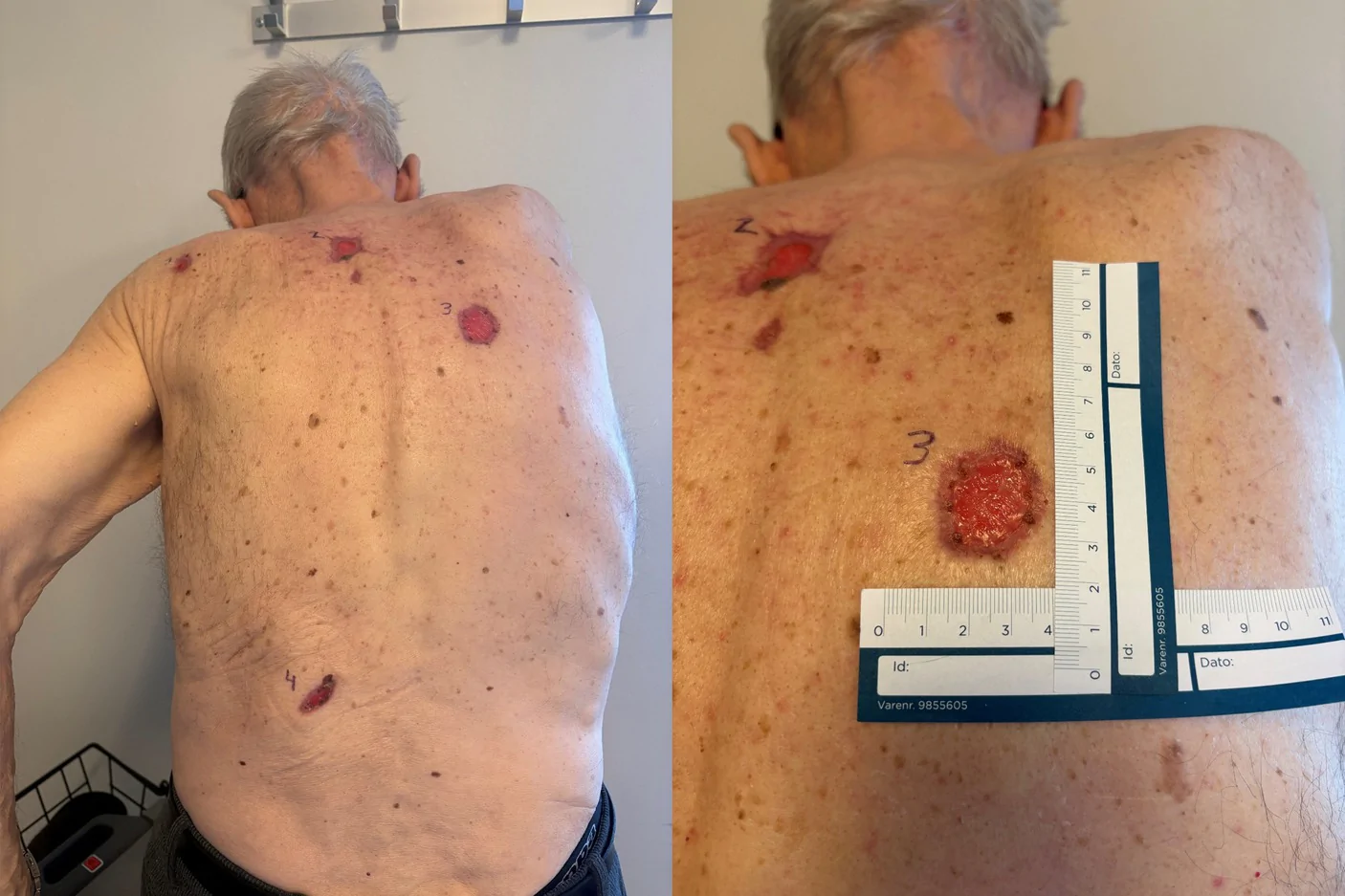
An overview photograph identifying the lesion of interest (right) should be accompanied by a close-up photograph including a measuring tape for scale (left).

The camera position must be at the same level as the skin lesion, with the skin lesion in the center of the photograph. The camera needs to move in a vertical direction and should not be pointed upward or downward (Figure 2). The anatomic region where the skin lesion is located should be included, not the entire region but enough to make the location identifiable to serve as an anatomic landmark. If the photograph is taken too close, the anatomic part may not be identified, and from a distance, the lesion will be difficult to identify (2). It is ideal to combine an overview photograph of the entire body region with a close-up photograph of the skin lesion. The distance between the camera and the skin lesion should be approximately 10–20 cm (21). The use of a measuring tape is beneficial in evaluating the size of a skin lesion (Figure 3).

#### Photography angles

Correct camera angle is fundamental to capturing informative medical photographs. The camera must be held perpendicular to the skin lesion of interest, and the lesion should be centered in the frame. Multiple shots from different angles may be required. Photographing the patient in the anatomical position is preferred unless the skin lesion of interest is hidden in that position.

**Figure 4:**
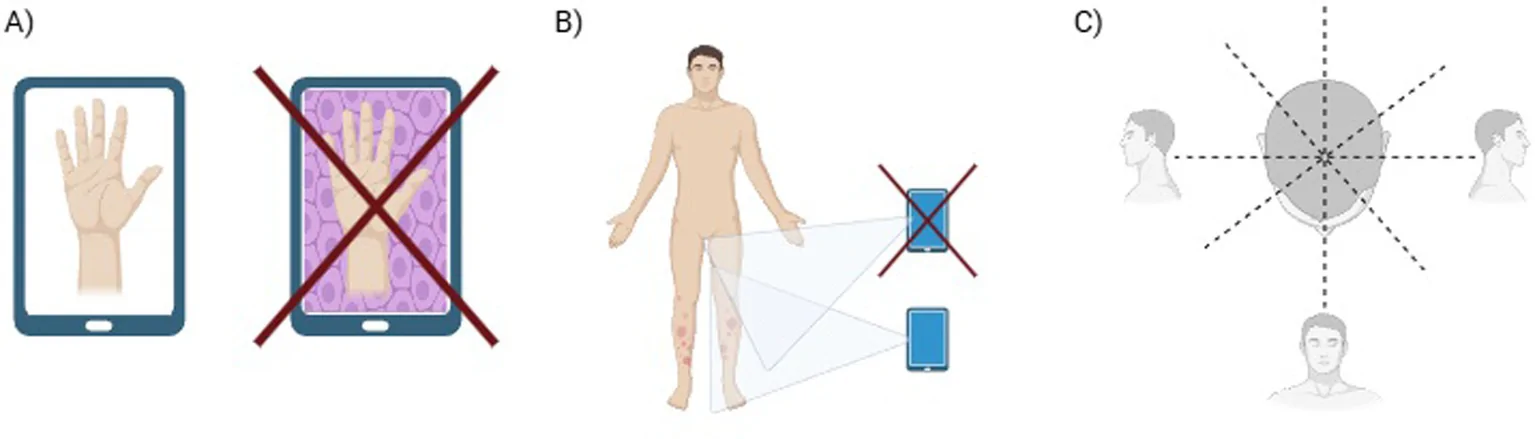
Three-step guide to optimal medical photography. **(A)** Use an even, neutral, non-reflective, monochrome background. **(B)** Position the camera perpendicular to and at the same level as the skin lesion; avoid upward or downward camera angles. **(C)** Multiple photographs from different angles are recommended. Photographing the patient in the anatomical position is recommended. Eight photography angles are suggested (created in BioRender).

Eight photography angles are important to know to correctly capture medical photography. Not all eight angles are required every time, as the necessary angles can be selected depending on the skin lesion of interest. In the frontal view, the patient’s body is directed toward the camera, and the patient is looking at the camera. In the oblique view (right and left), the patient’s whole body is turned 45° from the frontal view, and the patient is looking straight ahead, away from the camera. The lateral view (right and left) is when the patient’s whole body is rotated 90° from the frontal view. The same angles can be taken from the back with the posterior view and posterior oblique view (15, 16) (Figure 4).

### Photography of common non-neoplastic skin diseases

#### Acne and rosacea

To adequately capture photographs of acne or rosacea, at least a set of three photographs is recommended: front, right, and left 90° views (29). The 45° oblique view, with the entire body turning 45° from the front view and not just the face turning away from the camera, is useful to depict depth and contour in the documentation of acne scars (30). To document acne changes on the lower chin or neck, three more photographs, such as front, right, and left, will be needed of the neck, with the patient looking upward and the nose pointing toward the ceiling.

#### Alopecia

A set of five photographs is recommended to adequately capture alopecia. It is recommended to take photographs of the vertex from the top, lateral views from the right and left, and photographs from the front and back. An extra photograph from the front with the patient looking down, pointing the nose toward the floor, may be a useful supplement in cases of frontal alopecia (31, 32).

#### Atopic dermatitis and psoriasis

A photograph of each body region (head and neck, upper and lower extremities, and trunk and back) in the anatomic position is required. The photographs should be captured from all eight angles. A close-up photograph is recommended along with an overview photograph of the body region to evaluate the intensity of the skin lesions (12, 33–35).

#### Hidradenitis suppurativa

Photographs of areas with active hidradenitis suppurativa should be captured, including the head, neck, postauricular, axillae, chest, breasts, abdomen, groin, perineum, buttocks, and lower extremities. The camera should be positioned perpendicularly, capturing photographs of the axillae. A further 45 ° oblique photograph may help to distinguish between inflammatory nodules, abscesses, and draining tunnels. Photographs of the genital area are best captured when the patient is lying on the back on an examination table with their legs spread, and the camera is positioned perpendicularly to the genital area. Three sets of photographs of the nates are recommended when the perianal area is involved: a posterior photograph of the nates when the patient is standing, two photographs when the patient is lying on the side on an examination table, and one with and without spreading the nates with one hand. A close-up of the perianal area may also be useful (36, 37).

#### Urticaria, vitiligo, and exanthems

The affected area should be photographed. For full body involvement, it is recommended to capture a set of 10–20 photographs depending on the involved skin areas or body region.

Full-body photographs in all eight angles, including front, back, right, and left in 90° views, and 45° oblique views from the front and back, are recommended. The 45° oblique photographs are useful for evaluating induration, depth, and the degree to which the patches are raised. Anatomic position with the palms facing forward is recommended, as well as anterior trunk, flexed arms from anterior and posterior angles, axillae with both hands resting on the head, back, posterior legs, and side photograph in step position may be considered to visualize skin lesions on the inner thigh. Overview and close-up photographs are recommended for proper assessment (38, 39).

#### Genital warts, genital infections, lichen sclerosis, and other skin conditions involving the genital area

Three–four photographs are valuable for the genital area: two from above, with the patient lying on their back on an examination table with their legs together, including an overview and a close-up photograph; and one with the legs spread and the camera positioned perpendicular to the genital area or resting on the examination bed. A photograph of male patients lifting their testicles to visualize the perineum area may be beneficial. As for hidradenitis suppurativa, three photographs are recommended for perianal lesions: a posterior photograph of the nates in a standing position, and two photographs with the patient in a side position on an examination bed with and without spreading the nates with one hand (37).

### The future of dermatological photography

Imaging in dermatology goes beyond ordinary photography of skin diseases, and the future includes innovation in both equipment and techniques. Three-dimensional (3D) photography using simultaneous multi-camera imaging to capture and create a 3D reconstruction of the entire skin surface or a total-body photograph enables the creation of a 3D avatar that allows dermoscopic images to be mapped to specific lesions on the avatar and is gaining ground, especially in skin cancer clinics (40, 41).

Since the development of the Wood’s light in 1903, ultraviolet (UV) light has become an important tool in dermatology. Traditionally, UV imaging has been used to examine the skin to assess superficial cutaneous infections and pigmentary changes (42). UV fluorescent photography is a technique in which UV light is used to illuminate the skin, causing it to fluoresce and emit longer-wavelength light that is captured by the camera to reveal features not seen under normal lighting. UV fluorescent photography is useful for documenting vitiligo, acne (*Propionibacterium acnes* fluorescence), detecting pigmentation disorders, fungal infections (e.g., *Microsporum* species) (43), wound analysis, and assessing tumor margins of basal cell carcinomas (44, 45).

UV reflectance photography uses a camera with a UV-selective filter, allowing ultraviolet radiation to pass while blocking all other wavelengths of light from entering the sensor to capture images in the UV spectrum. This technique reveals superficial skin structures and pigment changes invisible to the naked eye, such as skin damage, photodamage, inflammation, and subtle pigmentary disorders such as vitiligo, and the effectiveness of sunscreen (46).

Infrared thermal imaging detects and visualizes infrared radiation produced by body tissue to capture temperature variations through specialized infrared cameras. This technology allows for identifying areas of increased or decreased thermal activity to assess inflammatory conditions, detect skin cancers, and assess skin hydration by measuring and visualizing it (47).

Automated skin lesion detection algorithms represent a promising future direction for enhancing the diagnostic utility of photographs. Huang et al. demonstrated that digital photographs can be computationally converted into narrowband-like spectral images using advanced AI and image-processing algorithms, resulting in better detection of skin lesions (48).

## Discussion and conclusion

Medical photography has become an integral part of daily clinical practice and patient care; therefore, tips and guidelines to improve clarity, lighting, positioning, exposure, background, angles, and scale are warranted. Three basic elements are essential for high-quality medical photography: resolution, lighting, and focus. Resolution is defined by the number of pixels, and a high-resolution photograph contains a large number of pixels, resulting in greater detail and sharper images. It is recommended to capture photographs in a well-lit environment or near a window with ample indirect natural light. The use of flash is generally not recommended but can be beneficial in some situations. Accurate focus is crucial; however, most cameras today have autofocus, which is adequate for medical photographs. Furthermore, three principles are important for accurately documenting a clinical condition and conveying information such as lesion location, size, color, texture, and depth: background, perspective, and angles. It is recommended to use an even, neutral, non-reflective, monochrome background, position the camera perpendicular to and at the same level as the skin lesion, and capture multiple images from different angles.

Several elements are important for standardizing serial clinical photographs used to monitor disease progression or treatment response over time. Whenever possible, the same camera device should be used. A consistent background is preferable, together with a fixed camera-to-subject distance and standardized framing, including the same patient position guided by anatomical landmarks. In addition, lighting conditions should be standardized, ideally by maintaining a consistent location and orientation relative to the window to ensure similar natural illumination across time points.

Computational photography algorithms employed by modern smartphones may influence the visual appearance of skin lesions. These processing steps have the potential to affect the fidelity of dermatological images by altering clinically relevant color and surface characteristics. Guidelines for standardizing photography should consider computational photography tools, including automatic adjustments of color balance, contrast, sharpness, noise reduction, high dynamic range, and skin texture enhancement are warranted.

### Ethical considerations

Obtaining informed consent prior to capturing a clinical photograph is essential, including providing information on the intended purpose, e.g., clinical care, education, or publication (49). Anonymization of photographs, by avoiding identifiable features such as the face, tattoos, jewelry, and birthmarks, is recommended (50). Photographs of sensitive anatomical sites such as the genital or breast regions require particular attention to consent, privacy, and secure handling. The use of personal devices for capturing photographs should be avoided; instead, only institution-approved systems and secure image storage should be used. All metadata, including GPS coordinates, date, and device information, should be removed prior to sharing or publication. Patients should also be instructed to adhere to the same privacy principles when sending a photograph or using teledermatology.
